# Construction of a set of novel and robust gene expression signatures predicting prostate cancer recurrence

**DOI:** 10.1002/1878-0261.12359

**Published:** 2018-08-11

**Authors:** Yanzhi Jiang, Wenjuan Mei, Yan Gu, Xiaozeng Lin, Lizhi He, Hui Zeng, Fengxiang Wei, Xinhong Wan, Huixiang Yang, Pierre Major, Damu Tang

**Affiliations:** ^1^ Department of Gastroenterology Xiangya Hospital Central South University Changsa Hunan China; ^2^ Division of Nephrology Department of Medicine McMaster University Hamilton Ontario Canada; ^3^ Father Sean O'Sullivan Research Institute Hamilton Canada; ^4^ The Hamilton Center for Kidney Research St. Joseph's Hospital Canada; ^5^ Department of Nephrology The First Affiliated Hospital of Nanchang University Jiangxi China; ^6^ Harvard Medical School and Massachusetts General Hospital Boston MA USA; ^7^ Department of Thoracic Surgery Fourth Hospital of Hebei Medical University Shijiazhuang City China; ^8^ The Genetics Laboratory Longgang District Maternity and Child Healthcare Hospital Shenzhen Guangdong China; ^9^ Division of Medical Oncology Department of Oncology McMaster University Hamilton Ontario Canada

**Keywords:** biomarkers, MUC1, prostate cancer, prostate cancer recurrence

## Abstract

We report here numerous novel genes and multiple new signatures which robustly predict prostate cancer (PC) recurrence. We extracted 696 differentially expressed genes relative to a reported PC signature from the TCGA dataset (*n* = 492) and built a 15‐gene signature (SigMuc1NW) using Elastic‐net with 10‐fold cross‐validation through analyzing their expressions at 1.5 standard deviation/SD below and 2 SD above a population mean. SigMuc1NW predicts biochemical recurrence (BCR) following surgery with 56.4% sensitivity, 72.6% specificity, and 63.24 median months disease free (MMDF) (*P* = 1.12e‐12). The prediction accuracy is improved with the use of SigMuc1NW's cutpoint (*P* = 3e‐15) and is further enhanced (sensitivity 67%, specificity 75.7%, MMDF 45.2, *P* = 0) when all 15 genes were analyzed through their cutpoints instead of their SDs. These genes individually associate with BCR using either SD or cutpoint as the cutoff points. Eight of 15 genes are individual risk factors after adjusting for age at diagnosis, Gleason score, surgical margin, and tumor stage. Eleven of 15 genes are novel to PC. SigMuc1NW discriminates BCR with time‐dependent AUC (tAUC) values of 76.6% at 11.5 months (76.6%–11.5 m), 73.8%‐22.3 m, 78.5%‐32.1 m, and 76.4%–48.4 m. SigMuc1NW is correlated with adverse features of PC, high Gleason scores (odds ratio/OR 1.48, *P* < 2e‐16), and advanced tumor stages (OR 1.33, *P* = 4.37e‐13). SigMuc1NW remains an independent risk factor of BCR (HR 2.44, 95% CI 1.53–3.87, *P* = 1.62e‐4) after adjusting for age at diagnosis, Gleason score, surgical margin, and tumor stage. In an independent PC (MSKCC) cohort (*n* = 140), these 15 genes were altered in PC vs normal tissue, metastatic PCs vs primary PCs, and recurrent PCs vs nonrecurrent PCs. Importantly, a 10‐gene subsignature SigMuc1NW1 predicts BCR in MSKCC (*P* = 3.11e‐15) and TCGA (*P* = 3.13e‐12); SigMuc1NW1 discriminates BCR at 18.4 m with tAUC as 82.5%. Collectively, our analyses support SigMuc1NW as a novel and robust signature in predicting BCR of PC.

**Abbreviations**
ADT: androgen deprivation therapy
BCR: biochemical recurrence
CRPC: castration‐resistant prostate cancer
DEGs: differentially expressed genes
DFS: disease‐free survival
GS: Gleason score
MMDF: median months disease free
OS: overall survival
PC: prostate cancer
RP: radical prostatectomy

## 1 Introduction

Prostate cancer (PC) is the most common malignancy in men in the developed countries (Ferlay *et al*., 2015). The disease progresses with a large degree of disparity. While a large proportion of the low grade [Gleason score 6/WHO grade (group) I or ISUP (the International Society of Urological pathology) grade 1] tumors are not life‐threatening, approximately 30% of patients after radical prostatectomy (RP) will experience disease recurrence with a rise in serum prostate‐specific antigen (PSA) (Zaorsky *et al*., 2013); this biochemical recurrence (BCR) indicates significantly increased risk for PC metastasis and castration‐resistant prostate cancer (CRPC) (Semenas *et al*., 2012). Metastasis is the leading cause of PC death. The standard treatment for metastatic PC is androgen deprivation therapy (ADT), which offers palliative care as resistance in the form of CRPC always occurs. In this regard, intervention at the point of BCR will be more effective than at time when PC has advanced to later stages. Thus, effectively assessing PCs with increased risk of BCR is highly desirable.

Recent developments have yielded three commercially available mRNA expression‐based multigene panels, Oncotype DX (Genomic Prostate Score/GPS), Prolaris (cell cycle progression/CCP), and Decipher (Genomic Classifier/GC). Both the 17‐gene Oncotype DX and the 31‐gene Prolaris improve risk stratification of patients with high risk of PC recurrence at time of diagnosis (Albala *et al*., 2016; Cuzick *et al*., 2011; Klein *et al*., 2014; Knezevic *et al*., 2013; Oderda *et al*., 2017) and after radical prostatectomy (RP) (Cooperberg *et al*., 2013; Cullen *et al*., 2015). The 22‐gene Decipher predicts metastasis following RP (Erho *et al*., 2013; Karnes *et al*., 2013; Klein *et al*., 2016). While these and other biomarkers assist decision making and thus improve patient management, their clinical application requires further validation (Lamy *et al*., 2017; Martin, 2016; McGrath *et al*., 2016; Patel and Gnanapragasam, 2016; Ross *et al*., 2016; Zhuang and Johnson, 2016). There is a clear need to improve our ability to stratify PCs with high risk of recurrence following RP. The challenge in accurately predicting PC recurrence is in part attributable to a complex network of pathways that drive the disease development.

The Mucin 1 (MUC1) network plays a role in BCR after RP (Eminaga *et al*., 2016; Lin *et al*., 2017). MUC1 is a tumor‐associated antigen that has been intensively investigated (Apostolopoulos *et al*., 2015; Kufe, 2009; Nath and Mukherjee, 2014). MUC1 is a glycoprotein that is expressed on the apical surface of most epithelial tissues (de Paula Peres *et al*., 2015; Wurz *et al*., 2014); its glycosylation is altered in over 70% of cancers (Kufe, 2009; de Paula Peres *et al*., 2015). In PC, MUC1 expression is upregulated and aberrantly glycosylated (Arai *et al*., 2005; Cozzi *et al*., 2005; Rabiau *et al*., 2009). These abnormalities are associated with angiogenesis (Papadopoulos *et al*., 2001) and adverse clinical features (Eminaga *et al*., 2016). MUC1 upregulation weakly correlates with shortening in disease‐free survival (DFS) and overall survival (OS) (Eminaga *et al*., 2016) and associates with adverse histopathology following RP (Durrani *et al*., 2015). A 3‐protein panel (AZGP1, MUC1, and p53) is related to poor prognosis in men with local PC (Severi *et al*., 2014). Increases in MUC1 mRNA expression were detected in metastatic PC. Genomic alterations in a 25‐gene MUC1 network were marginally associated with PC recurrence (Wong *et al*., 2016). Among these 25 genes, genomic alterations in nine genes substantially enhanced the association (Lin *et al*., 2017).

To further explore the biomarker value of the MUC1 network, we examined the transcriptome of the 9‐gene MUC1 genomic signature using the TCGA Provisional dataset within cBioPortal, and established 696 differentially expressed genes (DEGs). From these DEGs, a 15‐gene panel and multiple subpanels were constructed. These signatures robustly associate with reductions in DFS following RP in two independent PC datasets (*n* = 492 and *n* = 140). Cutpoints have been derived, which not only enhance the power of these signatures in the stratification of men with higher risk of BCR but also provide a guideline for the subsequent validation and clinical application. Taken together, we have constructed a set of novel and robust signatures to assess PC recurrence following RP.

## 2 Materials and methods

### 2.1 cBioPortal

The cBioPortal (Cerami *et al*., 2012; Gao *et al*., 2013) (http://www.cbioportal.org/index.do) database contains the most well‐organized and comprehensive data on cancer genetics for various cancer types. The TCGA Provisional datasets for individual cancer types cover genetic abnormalities, transcriptomes determined by either cDNA microarray or RNA sequencing, and the detailed clinical characteristics including disease outcomes (recurrence and mortality). The TCGA Provisional PC dataset has 492 patients with localized PC.

### 2.2 Establishing of multigene panel signatures

The largest TCGA Provisional dataset within the cBioPortal database (Cerami *et al*., 2012; Gao *et al*., 2013) (http://www.cbioportal.org/index.do), which includes 492 patients with follow‐up data, was used to derive 696 DEGs that are associated with the 9‐gene signature of the MUC1 genomic network (Lin *et al*., 2017). These DEGs were defined at *q* < 0.001. Follow‐up period, recurrence, and other clinical data were also extracted. Elastic‐net logistic regression within the glmnet package in R was used to select variables with major impacts on BCR with 10‐fold cross‐validation; the mixing parameter of Elastic‐net α was used at: 0.2 and 0.8. When α = 0, Elastic‐net operates as Ridge regression which does not perform covariate selection but shrink the coefficients of correlated predictors toward one another. When α = 1, it runs as Lasso which tends to select one covariate among a group of related covariates; this will make a signature less robust. To enhance selection of highly related variables as a group while maintaining the number of covariates to minimum, we used a range of α value: 0.2 and 0.8. With this system, a 15‐gene panel was selected.

### 2.3 Assignment of signature scores to patients/tumors

Individual component genes have been examined to predict BCR using univariate Cox proportional hazards (PH) regression; the Cox coefficients for individual component genes were obtained. The PH assumption was also determined. This analysis was performed using the R ‘survival’ package. The signature scores for individual patients were given using Sum (coef_1_ + coef_2_ + … … + coef_n_), where coef_1_ … coef_*n*_ are the coefs of individual genes.

### 2.4 Cutpoint estimation

Cutpoint of signature in separation of recurrent tumor from those without BCR was estimated using Maximally Selected Rank Statistics (the Maxstat package) in R. We also retrieved the RNA expression data for each component gene from the TCGA dataset; the cutpoints to discriminate PCs with BCR from those without BCR for each RNA expression data were also derived.

### 2.5 Regression analyses

Logistic regression was performed using R. Cox proportional hazards (Cox PH) regression analyses were carried out using the R survival package. The PH assumption was examined.

### 2.6 Pathway enrichment analysis

The GAGE (Luo *et al*., 2009) and Reactome (Yu and He, 2016) packages in R were used to analyze gene sets and pathways that were enriched in DEGs using the KEGG (Kyoto Encyclopedia of Genes and Genomes) and GO (gene ontology) databases.

### 2.7 Statistical analysis

Fisher's exact test was performed using the GraphPad Prism 5 software. Kaplan–Meier surviving curves and log‐rank test were carried out using the R survival package, and tools provided by cBioPortal. Univariate and multivariate Cox regression analyses were run using the R survival package. Time‐dependent receive operating characteristic (tROC) analysis was performed using the R timeROC package. A value of *P* < 0.05 is considered statistically significant.

## 3 Results

### 3.1 Identification of DEGs which are associated with the 9‐gene MUC1 genomic signature

**Figure 1:**
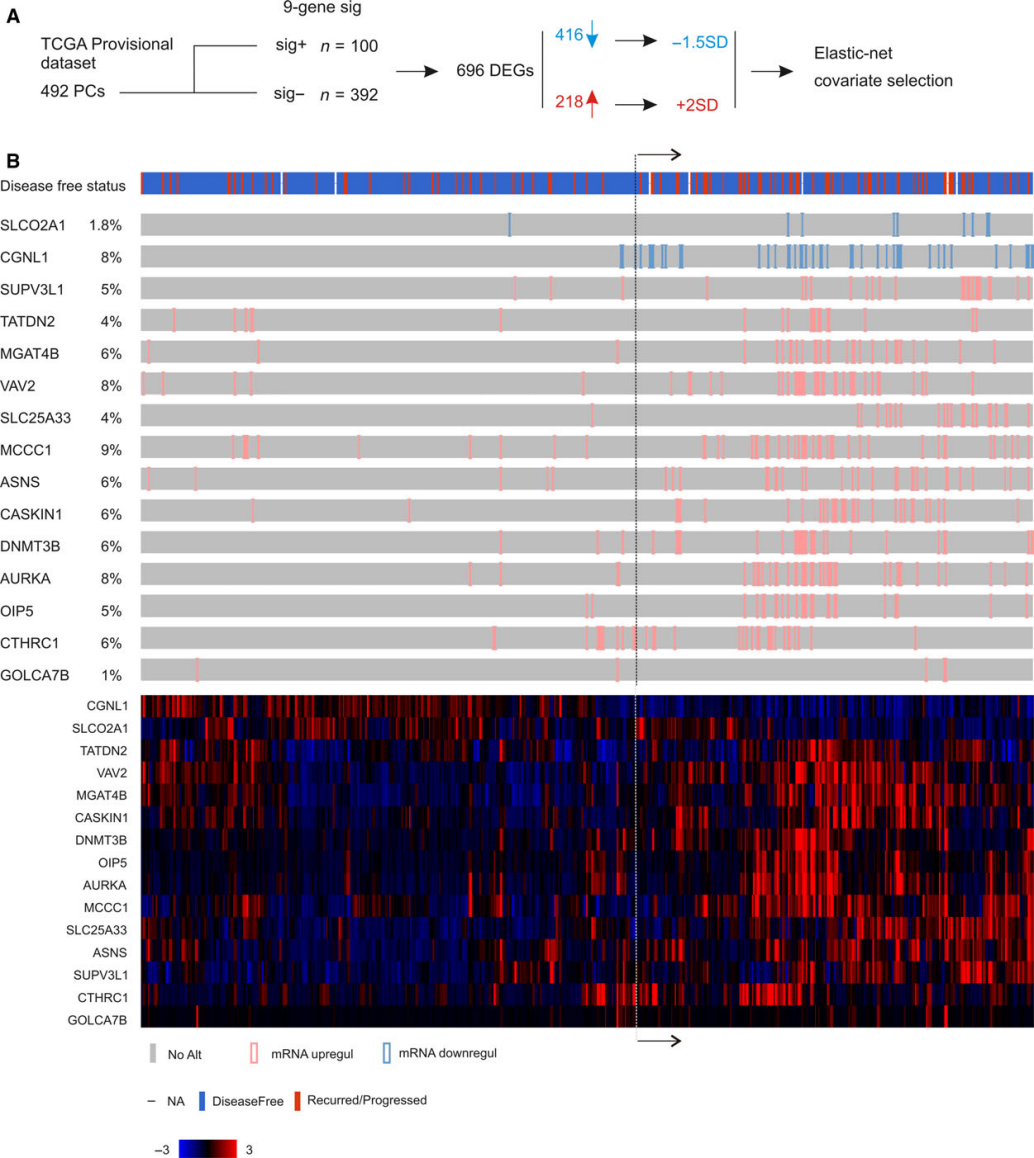
Construction of a 15‐gene signature. (A) Strategy used to produce the signature. The TCGA Provisional dataset within cBioPortal has 492 prostate cancers with gene expression profiled by RNA sequencing. The cohort was first divided into two populations: one (*n* = 100) positive for a 9‐gene signature derived from a MUC1 genomic network (Lin *et al*., 2017) and another (*n* = 392) negative for the signature. From these two populations, 696 differentially expressed genes (DEGs) were identified based on the mean mRNA expression and *q* < 0.001. These DEGs consist of 461 downregulated genes and 218 upregulated genes. For the downregulated genes, we have assigned tumors with gene expression at 1.5 SD (standard deviation) lower than a reference population mean (−1.5 SD); for those upregulated genes, we have located PCs with these gene expression at 2 SD above the population mean. We then performed model‐building using regularization‐coupled covariate selection of these 696 DEGs for their impact on BCR using the Elastic‐net penalty in the R glmnet package (Fig S1 for a typical selection), which resulted in a 15‐gene signature (SigMuc1NW). (B) PCs of the TCGA cohort with −1.5 SD (SLCP2A1 and CGNL1) and 2 SD expression are shown using OncoPrint (top gray illustration) and clustered (bottom color image). The disease‐free status is also included. The illustration was generated using tools provided by cBioPortal.

Biochemical recurrence (BCR) after surgical resection occurs in 30–40% of patients (Punnen *et al*., 2014); approximately 40% of these patients will develop metastatic disease (Briganti *et al*., 2015; Den *et al*., 2014). Improving our ability in predicting BCR risk is clearly critical in preventing metastatic progression. We have recently constructed a 9‐gene genomic signature from the MUC1 genomic network (Lin *et al*., 2017); the signature effectively predicts BCR using the TCGA Provisional dataset: sensitivity 34.8%, specificity 83.6%, and median months disease free (MMDF) 73.36 months (*P* = 5.57e‐5) (Lin *et al*., 2017). BCR is a complex process driven by multiple pathway alterations. In this regard, we reasoned that the transcriptome associated with the 9‐gene genomic signature may yield a better signature. To investigate this possibility, we analyzed the 9‐gene signature‐associated transcriptome using the TCGA Provisional dataset within the cBioPortal database following the strategy outlined in Fig. 1A. Among 492 patients/tumors, 100 were positive for the signature (Fig. 1A). A comparison to the mean expression of individual genes between these 100 PCs and other 392 PCs revealed a total of 696 differentially expressed genes (DEGs), which were defined at *q* < 0.001 (Table S1). These DEGs contained 416 downregulations and 280 upregulations (Fig. 1A; Table S1). Geneset enrichment analysis of these DEGs using the KEGG (kegg) kegg.set.hs dataset and Gaga package in R revealed the upregulation of the genesets regulating cell cycle, oocyte meiosis, progesterone‐mediated oocyte maturation (Table S2A), and downregulation of the genesets mediating focal adhesion and others (Table S2B). With the Gene Ontology (go) go.sets.hs dataset, the upregulated genesets include those regulating multiple aspects of cell cycle progression, DNA metabolism, and other processes related to cell proliferation (Table S2C). Downregulated genesets contain those that mediate cell adhesion, extracellular processes, and other events (Table S2D). Pathway enrichment analysis of the 696 DEGs using the Reactome package in R identified pathways regulating G1, M, DNA replication, and chromatid segregation (Table S2E). Collectively, the above analyses reveal an association of the 696 DEGs with PC cell proliferation, implying their potential in predicting PC progression.

### 3.2 Construction of a 15‐gene signature SigMuc1NW to predict BCR following radical prostatectomy (RP)

We then analyzed the contributions of these 696 DEGs to BCR using the TCGA Provisional cohort, in which the primary treatment was RP (cBioPortal). While the classic system to construct a signature is to randomly divide a dataset into a training set and testing set (Lin *et al*., 2017), we chose to use the system of cross‐validation. This system is selected due to our large number of DEGs to be assessed for their impact on BCR and the availability of the powerful machine learning programs in the glmnet R package. Based on the heterogeneity of PCs, we reasoned that these DEGs may affect BCR when their expression is beyond a threshold level. For the downregulated DEGs, we separated PCs with their expression lower than 1.5 SD (standard deviation) of a reference population mean from those without this level of downregulation. For the upregulated DEGs, we grouped PCs with DEG expressions above 2 SD from the reference population mean (Fig. 1A). A reference population was either tumors within the dataset that are diploid for the gene of interest or the intact tumor population (http://www.cbioportal.org/faq.jsp). The justifications of using the levels of −1.5 SD downregulation and 2 SD upregulation here were based on our publication (Ojo *et al*., 2017) and to maintain a sufficient number of DEGs available for variable selection as a value below −1.5 SD or above 2 SD significantly reduced the number of qualified DEGs (data not shown).

Using this re‐organized dataset containing the downregulations, upregulations, follow‐up period, and recurrence status for each patient, we then performed covariate selection with regularization using Elastic‐net logistic regression within the R glmnet package (Fig. 1A). To balance the selection of highly correlated covariates and minimization of the number of covariates, we ran Elastic‐net with the mixing parameter α set at 0.2 or 0.8. A 10‐fold cross‐validation was used in all selection settings. As expected, more covariates were selected at α = 0.2 (*n* = 17) than α = 0.8 (*n* = 5) (Fig. S1). We also performed covariate selection with a different setting (s = 0.5) which resulted in more covariates than the setting of α = 0.2. We then removed those DEGs with coefficient < 0.01 in the s = 0.5 setting and < 0.001 in the α = 0.2 setting. This resulted in a panel of 15 genes (SigMuc1NW; NW referring to network), including all 5 genes selected at α = 0.8, 14 genes selected from α = 0.2 (including all 5 genes selected at α = 0.8), and 15 DEGs from *s* = 0.5 (including all 14 genes selected at α = 0.2) (Table 1).

**Table 1:** The component genes of SigMuc1NW

Gene	Locus	Name	Role in PC/other tumorigenesis	References
SLCO2A1^a^	3q22.1‐q22.2	Solute carrier organic anion transporter family member 2A1	Unknown/inactivation of it facilitates color cancer formation	Guda *et al*., 2014;
CGNL1^a^	15q21.3	Cingulin like 1	Unknown/unknown	NA
SUPV3L1^b^	10q22.1	Suv3 like RNA helicase	Unknown/unknown	NA
TATDN2^b^	3p25.3	TatD DNase domain containing 2	Unknown/unknown	NA
MGAT4B^b^	5q35.3	Mannosyl (alpha‐1,3‐)‐glycoprotein β‐1,4‐N‐acetylglucosaminyltransferase, isozyme B	Unknown/upregulation in murine hepatocellular carcinoma	Blomme *et al*., 2013;
VAV2^b^	9q34.2	Vav guanine nucleotide exchange factor 2	An androgen receptor (AR) coactivator; enhancing AR signaling in PC/	Magani *et al*., 2017;
SLC25A33^b^	1p36.22	Solute carrier family 25 member 33	Unknown/a mitochondrial UTP carrier; contributing to IGF‐induced cell growth	Lyons *et al*., 2017;
MCCC1^b^	3q27.1	Methylcrotonyl‐CoA carboxylase 1	Unknown/gain of function was reported in oral squamous cell carcinoma	Ribeiro *et al*., 2014;
ASNS^b^	7q21.3	Asparagine synthetase	Contributing to CRPC/	Sircar *et al*., 2012;
CASKIN1^b^	16p13.3	CASK interacting protein 1	Unknown/unknown	NA
DNMT3B^b^	20q11.21	DNA methyltransferase 3 beta	Likely facilitating CRPC/	Gravina *et al*., 2011;
AURKA^b^	20q13.2	Aurora kinase A	Contributing to CRPC/	Mosquera *et al*., 2013;
OIP5^b^	15q15.1	Opa interacting protein 5	Unknown/a cancer testis antigen detected in colorectal cancer	Tarnowski *et al*., 2016;
CTHRC1^b^	8q22.3	Collagen triple helix repeat containing 1	Unknown/promoting tumorigenesis in multiple cancer types	Ke *et al*., 2014;
GOLGA7B^b^	10q24.2	Golgin A7 family member B	Unknown/unknown	NA

a −1.5 SD downregulated genes.

b 2 SD upregulated genes.

NA: not available.

Among the 15 genes, *SLCO2A1* and *CGNL1* are downregulated and the rest are upregulated (Table 1). Five genes *CGNL1, SUPV3L1, TATDN2, CASKIN1,* and *GOLGA7B* are of unknown functions in either prostate cancer tumorigenesis or tumorigenesis in general (Table 1). Six genes (*SLCO2A1, MGAT4B, SLC25A33, MCCC1, OIP5,* and *CTHRC1*) have been shown to affect the tumorigenesis of other cancer types but not PC (Blomme *et al*., 2013; Chen *et al*., 2013; Guda *et al*., 2014; Ke *et al*., 2014; Lyons *et al*., 2017; Ribeiro *et al*., 2014; Tarnowski *et al*., 2016) (Table 1). OIP5 (Opa interacting protein 5) is a cancer testis antigen and has been reported in other cancer types as a type of tumor‐associated antigen (TAA) (Tarnowski *et al*., 2016); its detection in PC here suggests OIP5 being a TAA for PC. The remaining four genes *VAV2* (VAV guanine nucleotide exchange factor 2), *ASNS* (asparagine synthesis), *DNMT3B* (DNA methyltransferase 3 beta), and *AURKA* (Aurora kinase A) not only all promote PC pathogenesis but also play a role in the development of CRPC (Gravina *et al*., 2011; Magani *et al*., 2017; Mosquera *et al*., 2013; Sircar *et al*., 2012). VAV2 is a coactivator of androgen receptor (AR) and sustains AR signaling under androgen deprivation therapy (ADT) (Magani *et al*., 2017); it also promotes angiogenesis and metastasis (Barrio‐Real and Kazanietz, 2012). AURKA plays a critical role in mitosis (Dominguez‐Brauer *et al*., 2015; Plotnikova *et al*., 2015) and promotes the development of neuroendocrine PC under ADT (Beltran *et al*., 2011; Mosquera *et al*., 2013). DNMT3B may regulate epigenetic events to facilitate CRPC development (Hoffmann *et al*., 2007). Collectively, evidence supports an association of SigMuc1NW with PC recurrence.

In line with this possibility, univariate Cox proportional hazards (PH) analysis revealed that all component genes at the defined level expression (−1.5 SD downregulation and 2 SD upregulation) significantly predict BCR (Table 2). Except for TATDN2 and OIP5, the PH assumption of the Cox model was confirmed. The prediction for some genes (*MGAT4B*,* ASNS*,* DNMT3B*, and *OIP5*) is robust (Table 2), particularly considering the prediction being individual gene‐based.

**Table 2:** Association of the component genes of SigMuc1NW with PC recurrence^a^

Genes	Coef^b^	HR^c^	95% CI^d^	*P*‐value
SLCO2A1^e^	1.5813	4.861	1.763–13.4	0.00225**
CGNL1^e^	0.9902	2.692	1.546–4.686	0.000464***
SUPV3L1^f^	0.8437	2.325	1.168–4.629	0.0163*
TATDN2^f^	1.3132	3.718	1.855–7.45	0.000213***
MGAT4B^f^	1.5178	4.562	2.245–9.272	2.73e‐5***
VAV2^f^	1.1027	3.012	1.671–5.429	0.000244***
SLC25A33^f^	1.096	2.992	1.55–5.777	0.00109**
MCCC1^f^	0.8336	2.302	1.322–4.007	0.00321**
ASNS^f^	1.3456	3.84	2.064–7.145	2.15e‐5***
CASKIN1^f^	1.0286	2.797	1.55–5.047	0.000636***
DNMT3B^f^	1.2919	3.64	1.928–6.87	6.73e‐5***
AURKA^f^	1.0966	2.994	1.692–5.298	0.000166***
OIP5^f^	1.365	3.914	2.022–7.576	5.13e‐5***
CTHRC1^f^	0.7981	2.221	1.15–4.289	0.0174*
GOLGA7B^f^	2.0406	7.695	2.388–24.79	0.00063***

a Univariate Cox analysis was performed using the TCGA Provisional cohort (*n* = 492).

b Cox coefficient.

c Hazard ratio.

d Confidence interval.

e Gene expression was < −1.5 SD of the reference population mean.

f Gene expression was at > 2 SD of the reference population mean.

*P < 0.05; **P < 0.01; ***P < 0.001.

**Figure 2:**
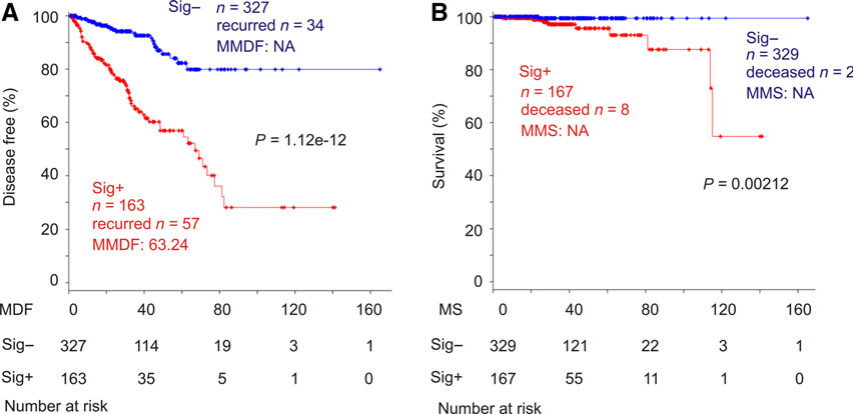
SigMuc1NW is associated with reductions in disease‐free survival (DFS) and overall survival (OS) in patients with PC. The TCGA Provisional cohort was used in these analyses. (A) The effect of SigMuc1NW on DFS. MDF: months disease free; MS: months survival; MMDF: median months disease free; NA: not available as MMDF being not reached. Numbers of patient at risk at the start of the indicated follow‐up period were included. (B) The impact of SigMuc1NW on OS. MMS: median months survival. Kaplan–Meier and log‐rank test were performed using the R survival Package.

In support of our selection of related genes, changes in the 15 genes show an overlapping profile (Fig. 1B, up panel) and their expression can be clustered (Fig. 1B, bottom panel). The downregulation/upregulation‐based alterations and gene expression‐derived cluster are well matched (Fig. 1B), providing a validation for our covariate selection. Importantly, patients with these changes are at risk of developing recurrent PC; that is, these patients are enriched with recurrent tumors (Fig. 1B, see the ‘Disease‐free status’ illustration). Tumors positive to SigMuc1NW are also robustly associated with reductions in disease‐free survival (DFS) (Fig. 2A, *P* = 1.12e‐12). The association has a sensitivity of 56.4% and specificity of 72.6%, which are significantly improved from the initially reported 9‐gene signature (sensitivity of 34.8%, specificity of 83.6%, *P* = 5.57e‐5) (Lin *et al*., 2017). Considering the TCGA cohort had 10 total mortality, it is intriguing that 8 of these 10 deaths occurred in patients with SigMuc1NW‐positive PC (Fig. 2B, *P* = 0.00212), which are consistent with VAV2, ASNS, DNMT3B, and AURKA being factors promoting CRPC development (Gravina *et al*., 2011; Magani *et al*., 2017; Mosquera *et al*., 2013; Sircar *et al*., 2012). As expected, SigMuc1NW displays an overlapping pattern with the 9‐gene genomic signature used to select DEGs (Fig. S2). Inclusion of SigMuc1NW substantially enhanced the association of the 9‐gene signature with BCR (Fig. S3A,C) and significantly correlates with a reduction in overall survival (OS) (Fig. S3B).

### 3.3 SigMuc1NW effectively discriminates recurrent PCs from those without BCR

**Figure 3:**
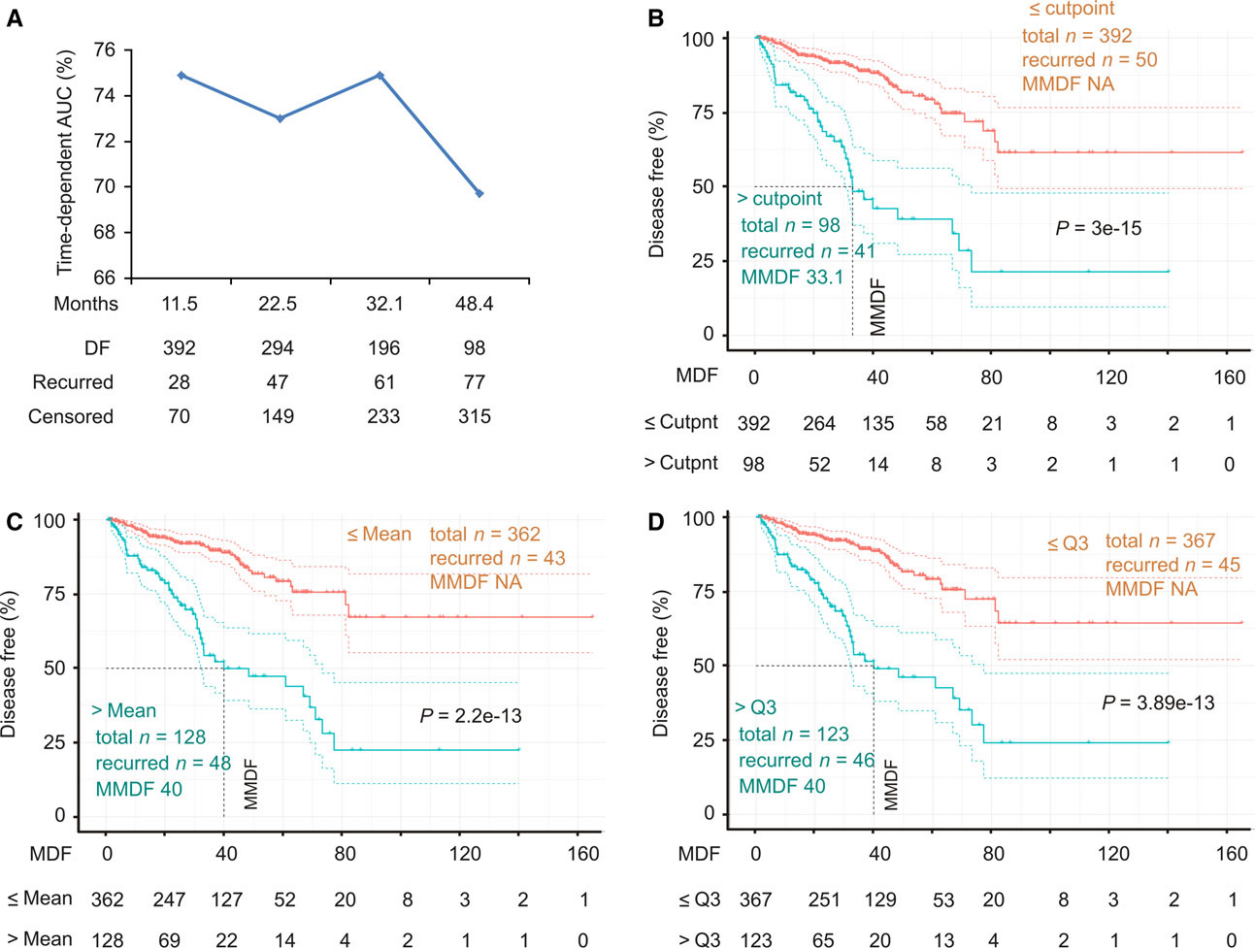
SigMuc1NW scores effectively stratify PCs with a high risk of recurrence. (A) All tumors within the TCGA Provisional cohort were scored for SigMuc1NW (see Results for details). The scores were analyzed for discrimination of tumors with high risk of recurrence using tROC. AUC at the indicated period of time (tAUC) along with the status of disease recurrence are indicated. DF: disease free. (B) The cutpoint of SigMuc1NW scores for effectively separating PCs with high risk of recurrence from low risk PCs was estimated (Fig S4 for details), followed by assigning binary codes to tumors based on the cutpoint (see Results for details). The effects of cutpoint on DFS of the patients in the TCGA cohort were then determined. (C, D) The effects of Mean and Q3 scores of SigMuc1NW on BCR in PC patients in the TCGA Provisional cohort. Kaplan–Meier and log‐rank test were performed using the R survival Package. The vertical dot line shows MMDF. The color dot curves are for 95% CI.

To examine the effectiveness of SigMuc1NW in separation of recurrent PC from those without BCR, we have assigned the alterations of the 15 genes with their Cox efficient (Table 2). The cumulative scores of SigMuc1NW for individual patients were then calculated as ∑(*f*_i_)_*n*_ (*f*_i_: Cox coefficient of gene_i_, *n* = 15) (Table S3). The sensitivity and specificity of the scores derived from SigMuc1NW in discrimination of BCR was analyzed using time‐dependent ROC (tROC). The scores discriminate recurrent PC with tAUC (area under curve) ranging from 74.9% at 11.5 and 32.1 months to 69.7% at 48.4 months (Fig. 3A), revealing SigMuc1NW being particularly effective in predicting earlier BCR. To further investigate this application, we determined the cutpoint of the SigMuc1NW scores in the separation of recurrent from nonrecurrent PC using Maximally Selected Rank Statistics using the Maxstat package in R (Fig. S4) and converted the scores into binary code; scores ≤ 1.7833 (cutpoint, Fig. S4) were assigned ‘0’ and scores > 1.7833 were assigned ‘1’. PCs with scores above the cutpoint have a dynamically faster profile of BCR than those with scores not above the cutpoint (Fig. 3B). Intriguingly, the cutpoint‐positive tumors even developed BCR in a shorter time frame (Fig. 3B; MMDF 33.1, 95% CI 30.9–73.4) compared to SigMuc1NW‐positive PCs (Fig. 2A; MMDF 63.2, 95% CI 40–77.3). The cutpoint thus not only will facilitate clinical examination of SigMuc1NW but also enhances its predictive power. Additionally, both mean and quartile 3 (Q3) scores can stratify patients with high risk of BCR with comparable effectiveness as SigMuc1NW (comparing Fig. 3C,D to Fig. 2A). Both mean and Q3 scores cover 48 and 46 recurrent PCs, respectively (Fig. 3C,D) which are more than the 41 recurrent PCs marked by the cutpoint (Fig. 3A). Thus, the mean (0.918), Q3 (1.019), and cutpoint (1.7883) scores can also be used to predict BCR following RP with a range of BCR risk. We further demonstrated SigMuc1NW (1.62e‐4), cutpoint (*P* = 2.05e‐5) (Table 3), Mean (*P* = 1.19e‐4), and Q3 (*P* = 1.67e‐4) (data not shown) being independent risk factors for PC recurrence after adjusting for age at diagnosis, RP Gleason scores, surgical margin, and TMN tumor stage. When the World Health Organization (WHO) PC grading system [WHO grade (group) I‐V] or its equivalent ISUP (the International Society of Urological Pathology) grade (Egevad *et al*., 2016; Gordetsky and Epstein, 2016) (Table S3 for details) instead of Gleason grade was used, SigMuc1NW (*P* = 2.05e‐4), cutpoint (*P* = 1.91e‐5), Mean (*P* = 1.37e‐4), and Q3 (*P* = 1.86e‐4) remain an independent risk factor for BCR. The demographics of the TCGA dataset with respect to the clinical characteristics used in the above multivariate Cox analyses are included (Table S4).

**Table 3:** Univariate and multivariate Cox analysis of SigMuc1NW for PC recurrence

Factors	Univariate Cox analysis			Multivariate Cox analysis			Multivariate Cox analysis		
	HR	95% CI	*P*‐value	HR	95% CI	*P*‐value	HR	95% CI	*P*‐value
Sig^a^	4.16	2.74–6.36	5.54e‐11*	2.44	1.53–3.87	1.62e‐4*	NA	NA	NA
Cutpoint^b^	4.6	3.03–6.97	6.44e‐13*	NA	NA	NA	2.67	1.70–4.20	2.05e‐5*
Age^c^	1.03	0.99–1.06	0.0981	0.999	0.97–1.03	0.9711	1.001	0.97–1.03	0.9756
GS^d^	2.19	1.76–2.72	1.49e‐12*	1.62	1.25‐2.11	2.71e‐4*	1.62	1.25–2.10	2.86e–4*
SMargin^e^	2.25	1.48–3.41	0.000137*	1.25	0.79–1.98	0.3306	1.28	0.81–2.02	0.2976
TumStge^f^	3.68	2.08–6.51	8.19e‐6*	1.82	0.97–3.40	0.0614	1.82	0.96–3.45	0.0668

a SigMuc1NW.

b SigMuc1NW‐derived cutpoint.

c Age at diagnosis.

d Radical prostatectomy Gleason score.

e Surgical margin.

f Tumor stages (0 for ≤ T2; 1 for T3 and T4).

HR, hazard ratio; CI, confidence interval; NA, not available.

*P < 0.05.

### 3.4 Enhancing the predictive efficiency of SigMuc1NW

**Figure 4:**
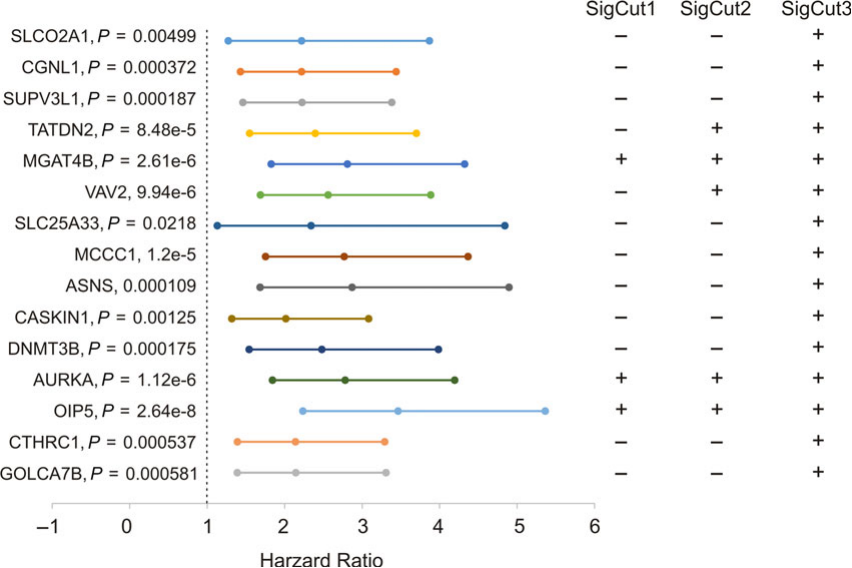
All 15 component genes are significantly associated with PC recurrence and the formulation of three subsignatures. The mRNA expression data for the 15 genes were retrieved from the TCGA Provisional dataset (cBioPortal). Individual cutpoints were derived, and binary codes were assigned to all tumors. The hazard ratio (HR) of PC recurrence for all individual genes was determined using the univariate Cox proportional hazards (PH) mode. The PH assumption was evaluated and confirmed. These analyses were carried out using the R survival package. Individual HR, the 95% CI, and *P*‐value are included. The inclusion of component genes in SigCut1, SigCut2, and SigCut3 were shown, which was based on the *P*‐values.

To further demonstrate SigMuc1NW being effective and robust, we analyzed the signature using the actual gene expression data instead of using SD (standard deviation)‐based distribution. For this purpose, the RNA sequencing data for all 15 SigMuc1NW genes were retrieved from the TCGA dataset and estimated for cutpoints in separating recurrent PCs (Table 4). All tumors were given a binary code for all 15 genes as described above with exception for both downregulated genes SLCO2A1 and CGNL1 in which tumors with expression less than the cutpoint were assigned ‘1’. Univariate Cox PH analysis was carried out with the PH assumption confirmed for all genes. All 15 genes, as defined by their cutpoint, significantly predict BCR (Fig. 4). Additionally, SLCO2A1, SUPV3L1, TATDN2, MGAT4B, VAV2, SLC25A33, ASNS, and OIP5 remain as independent risk factors of BCR after adjusting for age at diagnosis, RP Gleason scores, surgical margin, and TMN tumor stage (Table 5). These observations are appealing considering their single gene‐based nature, and that 8/15 component genes of SigMuc1NW possesses independent predicting value to BCR, which further supports SigMuc1NW as a signature for BCR.

**Table 4:** SigMuc1NW^a^ component genes defined at their cutpoints associate with BCR

Genes	Cutpoint^b^	*P*‐value	Coef^c^	*P*‐value
SLCO2A1	497.3292	0.09128	0.7967	0.00499**
CGNL1	3066.229	0.004126**	0.7966	0.000372***
SUPV3L1	545.8928	0.007953**	0.7992	0.000187***
TATDN2	1756.057	0.002471**	0.8731^d^	8.48e‐5***
MGAT4B	1818.718	6.389e‐5***	1.0331	2.61e‐6***
VAV2	1489.06	0.000547***	0.9402	9.94e‐6***
SLC25A33	297.5508	0.2522	0.8503	0.0218*
MCCC1	1233.159	0.001077**	1.0179	1.2e‐5***
ASNS	1041.086	0.01123*	1.0544	0.000109***
CASKIN1	106.4046	0.02646*	0.7006	0.00125**
DNMT3B	61.4086	0.008576**	0.9082	0.000175***
AURKA	81.1249	3.807e‐5***	1.0223	1.12e‐6***
OIP5	16.4317	4.237e‐7***	1.242^d^	2.64e‐8***
CTHRC1	180.8622	0.01389*	0.7608	0.000537***
GOLGA7B	23.2022	0.01249*	0.7623	0.000581***

a RNA sequencing data of SigMuc1NW's component genes were retrieved from the TCGA Provisional dataset (cBioPortal).

b Cutpoint was estimated using Maximally Selected Rank Statistics in R.

c Coefficient to BCR was determined using univariate Cox proportion hazard analysis.

d PH assumption was at *P* < 0.05.

*P < 0.05; **P < 0.01; ***P < 0.001.

**Table 5:** Univariate and multivariate Cox analysis of SigMuc1NW component genes defined at cutpoint for PC recurrence

Factors	Univariate Cox analysis			Multivariate Cox analysis		
	HR	95% CI	*P*‐value	HR	95% CI	*P*‐value
Age^a^	1.03	0.99–1.06	0.0981			NS^e^
GS^b^	2.19	1.76–2.72	1.49e‐12*	1.71–1.89^f^	(1.32–1.46)–(2.20‐2.41)^f^	4.48e‐7*–1.4e‐5*,^f^
SMargin^c^	2.25	1.48–3.41	0.000137*			NS^e^
TumStge^d^	3.68	2.08–6.51	8.19e‐6*	1.62–2.07	(0.85–1.08)‐(3.08–3.96)^f^	0.0272*,^h^–0.139^f^,^g^
SLCO2A1	2.22	1.27–3.87	0.00499*	1.82	1.04–3.19	0.0369*
SUPV3L1	2.22	1.46–3.38	1.87e‐4*	2.08	1.36–3.19	7.98e‐4*
TATDN2	2.39	1.55–3.70	8.48e‐5*	2.15	1.37–3.37	8.35e‐4*
MGAT4B	2.81	1.83–4.32	2.61e‐6*	1.77	1.23–2.78	0.0128*
VAV2	2.56	1.69–3.89	9.94e‐6*	1.93	1.26–2.95	0.0024*
SLC25A33	2.34	1.13–4.84	0.0218*	2.25	1.08–4.67	0.0297*
ASNS	2.87	1.68–4.90	1.09e‐4*	1.91	1.09–3.36	0.0239*
OIP5	3.46	2.24–5.36	2.64e‐8*	1.94	1.20–3.12	0.00638*

a Age at diagnosis.

b Radical prostatectomy Gleason score.

c Surgical margin.

d Tumor stages (0 for ≤ T2; 1 for T3 and T4).

e Not significant.

f Range of HR, 95% CI, and *P*‐values resulted from multivariate Cox analysis with the individual genes.

g The *P*‐values for SLCO2A1 (*P* = 0.0749), MGAT4B (*P* = 0.0891), ASNS (*P* = 0.0917), and OIP5 (*P* = 0.139).

^h^The *P*‐values for SUPV3L1 (*P* = 0.0431*), TATDN2 (*P* = 0.0272*), VAV2 (*P* = 0.0364*), and SLC25A33 (*P* = 0.0334*).

HR, hazard ratio; CI, confidence interval.

**Figure 5:**
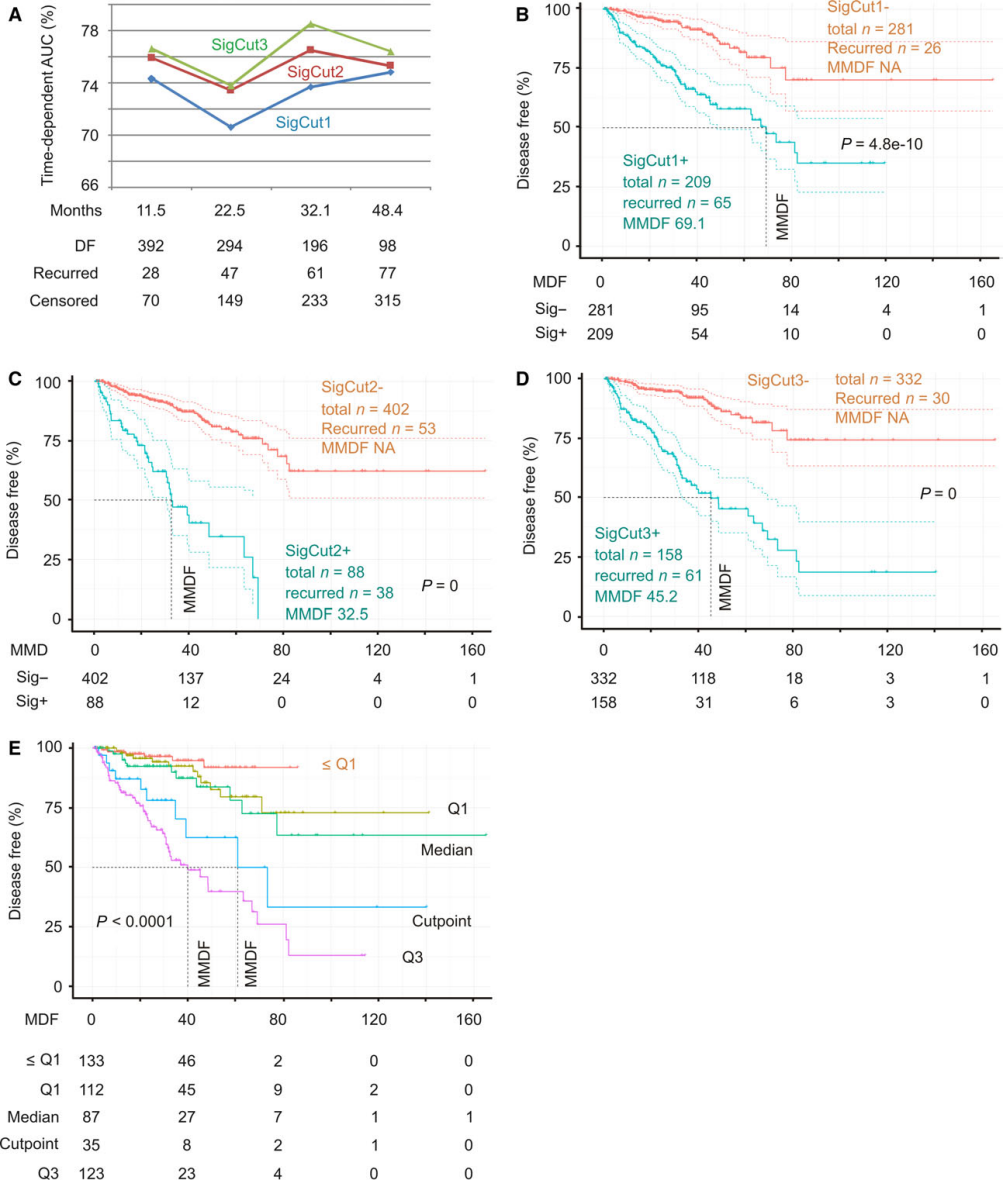
Analyses of SigCut1, SigCut2, and SigCut3 for their association with reductions in DFS. The TCGA Provisional dataset was used here. (A) All tumors were scored for SigCut1, SigCut2, and SigCut3 using the respective Cox coefficient. Time‐dependent AUCs for individual signature at the current follow‐up period and the corresponding recurrent status are shown. (B‐D) The associations of SigCut1, SigCut2, and SigCut3 with BCR. (E) The Q1, Median, Cutpoint, and Q3 scores of SigCut3 were analyzed for the stratification of PC with high risk of recurrence. The number of risk individuals at the indicated follow‐up period is included. The multiple Kaplan–Meier curves and log‐rank test were performed using the R survival package.

Using the Cox coefficients (Table 4), all cutpoint‐positive events were converted to the respective coefficient values (Table S5). Based on the robustness defined by *P*‐values (Fig. 4), we formulated three subsignatures SigCut1, SigCut2, and SigCut3 (Fig. 4). All tumors were then scored for SigCut1, SigCut2, and SigCut3 using ∑(*f*_i_)_*n*_ (*f*_i_: Cox coefficient of gene_i_, *n* = 3, 6, or 15). All three subsignatures discriminate recurrent PC effectively with tAUC > 70% (Fig. 5A). The respective cutpoints were determined: 1.0331/*P* = 6.166e‐8 for SigCut1, 4.0135/*P* = 1.005e‐11 for SigCut2, and 5.4067/*P* = 7.97e‐15 for SigCut3. The respective binary code for individual subsignature was then assigned to all tumors, which was used to perform survival analysis. All three subsignatures dramatically associate with reductions in DFS with SigCut2 and SigCut3 being more robust (Fig. 5B–D). Nonetheless, they predict BCR with a range of effectiveness in terms of the number of recurrent tumors included, the duration of MMDF, and sensitivity/specificity: 71.4%/63.9% for SigCut1, 41.8%/87.5% for SigCut2, and 67.7%/75.7% for SigCut3 (Fig. 5B–D). These three subsignatures can thus be used together to predict recurrent PCs; this will significantly enhance their predictive power.

The Q1 (1.647), Median (3.589), and Q3 (6.386) scores all effectively stratify PC with high risk of BCR with a range of effectiveness in terms of sensitivity/specificity/MMDF (median month disease free)/*P*‐value being 93.4%/31.8%/81.2/6.76e‐6 for Q1, 80.2%/56.9%/66.9/6.73e‐11 for Median, and 56%/82%/40/0 for Q3 (Fig. S5). When Q1, Median, Q3, and cutpoint of SigCut3 are used together, it offers an impressive system to stratify recurrent and nonrecurrent PCs with only a few recurrent cases in tumors with score < Q1 (Fig. 5E).

Furthermore, in comparison with SD‐defined SigMuc1NW (Fig. 2A), SigCut3 is clearly more effective (Fig. 5D). After adjusting for age at diagnosis, RP Gleason scores, surgical margin, and TMN tumor stage, SigCut1 (*P* = 0.00308), SigCut2 (*P* = 1.55e‐5), and SigCut3 (*P* = 2.97e‐6) independently predict BCR, respectively. All three signatures are associated with adverse features of PC: high tumor stages (T3 and T4) at odds ratio/95% CI of 1.78/1.51–2.12 (*P* = 2.39e‐11) for SigCut1, 1.55/1.37–1.77 (*P* = 1.33e‐11) for SigCut2, and 1.33/1.23–1.44 (*P* = 8.47e‐13) as well as for Gleason scores (8–10) at the respective odds ratio/95% CI of 2.19/1.86–2.6 (*P* < 2e‐16), 1.84/1.62–2.1 (*P* < 2e‐16), and 1.48/1.37–1.61 (*P* < 2e‐16). Taken together, these observations validate the efficacy of SigMuc1NW.

### 3.5 Validation of SigMuc1NW

**Figure 6:**
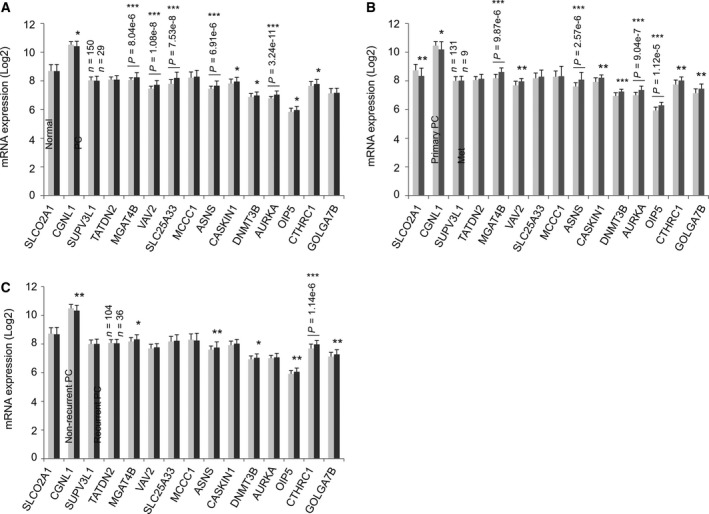
Alterations in the expression of the component genes in an independent PC population. Gene expression data determined by microarray were extracted from the MSKCC dataset (Robinson *et al*., 2015) within cBioPortal. The mRNA levels in normal and PC tissues (A), in primary PC and metastatic PC (B), and in nonrecurrent and recurrent PC (C) were determined. The number of cases used in the comparisons is indicated. Means ± SD are graphed. Statistical analyses were performed using Student's t‐test (2‐tailed). *P < 0.05, **P < 0.01, and ***P < 0.001.

We have made an effort to determine the individual component gene expression in PCs. The MKSCC (Cancer Cell 2010) (Taylor *et al*., 2010) dataset within cBioPortal has 216 PCs/patients with mRNA expression profiled using microarray; the expression data were organized for comparison between normal prostate tissues and PC (cBioPortal). Importantly, all primary PCs have been treated and the follow‐up information is available; this cohort thus supports survival analysis. To further validate SigMuc1NW constructed using RNA sequencing data from the TCGA Provisional dataset, mRNA expression data for all 15 component genes along with all clinical information were extracted from the MKSCC dataset. Tissues can be grouped into normal prostate (*n* = 29), primary PCs (*n* = 149), recurred PCs (*n* = 36), and metastatic PCs (*n* = 9) (cBioPortal). Using this setting, we demonstrated significant reductions of CGNL1 in primary PCs over normal prostate tissues, in metastatic PCs compared to localized PCs, and in recurrent PCs compared to nonrecurrent PCs among the two downregulated genes (SLCO2A1 and CGNL1) of SigMuc1NW (Fig. 6A–C). Significantly higher levels for most upregulated genes identified in SigMuc1NW were shown in the above comparisons (Fig. 6A–C), supporting the authenticity of SigMuc1NW.

**Figure 7:**
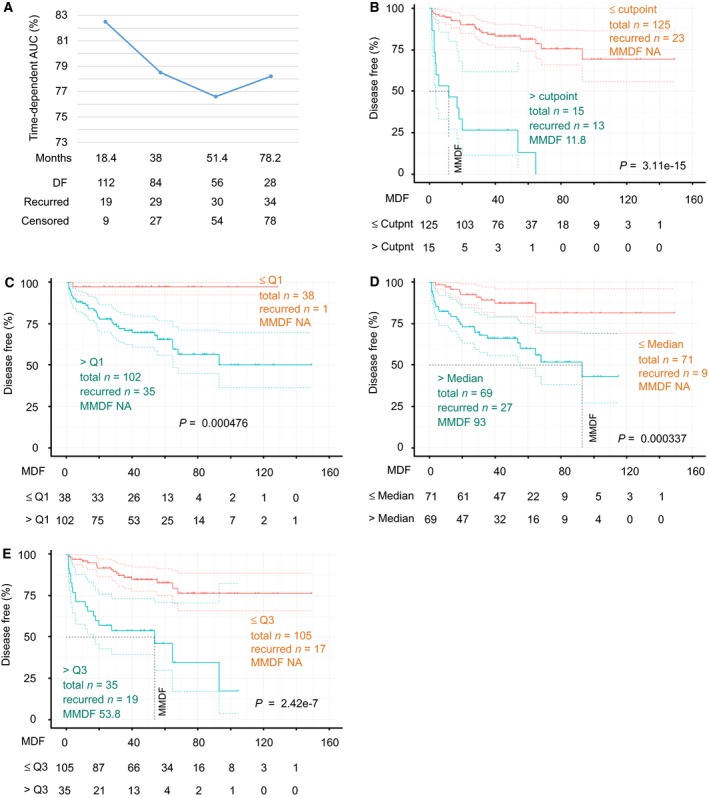
SigMuc1NW1 robustly predicts PC recurrent in an independent PC dataset. The follow‐up data along with mRNA expression data for all 15 genes were retrieved from the MSKCC dataset (Robinson *et al*., 2015). SigMuc1NW1 was formed using 10 genes (see Results for details). Time‐dependent AUCs were derived (A). The stratification of PC with increased risk of recurrence was analyzed using the cutpoint (B), Q1 (C), Median (D), and Q3 (E) scores of SigCut1NW1. Numbers of risk individuals at the current follow‐up period are also included.

**Figure 8:**
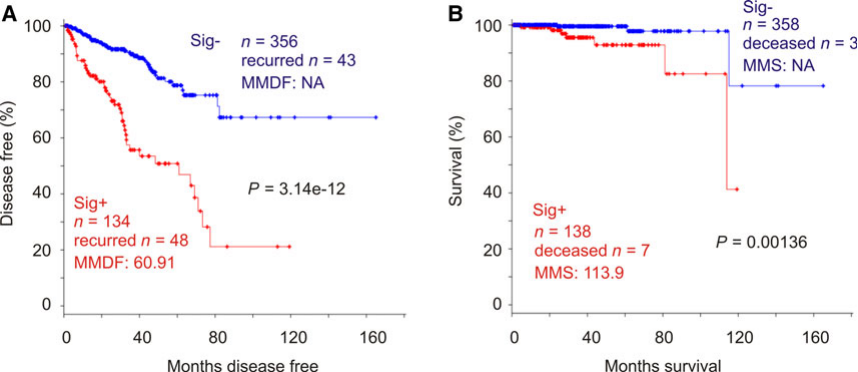
SigMuc1NW1 significantly correlates with reductions in DFS and OS in PC patients. The analyses were performed using the TCGA Provisional dataset. SigMuc1NW1 gene expression was based on the SD levels. Kaplan–Meier curve and log‐rank test were performed using tools provided by cBioPortal.

Following our system described above, cutpoints for all 15 genes were estimated, binary codes were assigned, and association of individual genes with BCR was determined using Cox PH regression (Table 6). Except MCCC1 being reversely associated with DFS and four genes without a significant correlation with DFS, other 10 genes significantly or robustly (CGNL1 and CTHRC1) predict BCR risk (Table 6). We then formulated a subsignature with these 10 genes (SigMuc1NW1). As described above, all tumors were scored for SigMuc1NW1 using their coefficients (Table 6). Analysis with tROC shows tAUC values being from 76.6% to 82.5% (Fig. 7A). SigMuc1NW1 thus effectively discriminates recurred PCs from nonrecurrent tumors across all follow‐up period from 18.4 months to 65 months (Fig. 7A); this efficiency matches that of SigMuc1NW in the discrimination of recurrent PCs in the TCGA cohort (Fig. 5A). Additionally, using the binary code derived from Q1 (0), Median (1.805), Q3 (3.727), and cutpoint (6.2136) scores of SigMuc1NW1, all these classifications significantly stratify recurrent PCs (Fig. 7B–E). The respective sensitivity/specificity/PPV (positive predictive value) are 36.1%/98.1%/86.7% for cutpoint, 97.2%/35.6%/34.3% for Q1, 75%/59.6%/39.1% for Median, and 52.8%/84.6%/54.3% for Q3 (Fig. 7B–E). The PPV for cutpoint is robust (86.7%). Collectively, through combination of Q1, Median, Q3, and cutpoint, PC recurrence could be effectively predicted for patients in the MSKCC cohort. The similar situation was also demonstrated in the TCGA cohort using SigMuc1NW. In a reverse validation effort, we demonstrated that SigMuc1NW1 is also robustly associated with BCR in the TCGA cohort and significantly correlates with a reduction in OS in the TCGA dataset (Fig. 8A,B). Taken together, we provide a thorough validation of SigMuc1NW and SigMuc1NW1.

**Table 6:** Cutpoint and Cox coefficients of SigMuc1NW component genes in the MSKCC cohort^a^

Genes	Cutpoint^b^	*P*‐value	Coef^c^	HR	95% CI	*P*‐value
SLCO2A1	8.155098	0.7073	0.6364	1.89	0.7835–4.558	0.157
CGNL1	10.02132	0.004758**	1.4679	4.34	2.084–9.038	8.8e‐5***
SUPV3L1	7.655546	0.7029	−0.6931	0.5	0.2277–1.098	0.0841
TATDN2	7.755133	0.969	−0.5149	0.5976	0.2476–1.442	0.252
MGAT4B	8.536576	0.01469*	1.3245	3.76	1.833–7.712	0.000302***
VAV2	7.801308	0.2076	0.8258	2.284	1.184–4.405	0.0138*
SLC25A33	8.653056	1	0.4752	1.608	0.6248–4.14	0.325
MCCC1	7.789343	0.2982	−1.0768	0.3407	0.1467–0.7911	0.0122*
ASNS	7.946625	0.01918*	1.1815	3.259	1.567–6.78	0.00157**
CASKIN1	8.142854	0.04935*	1.0985	3	1.529–5.886	0.0014**
DNMT3B	7.199673	0.06077	1.0373	2.822	1.385–5.749	0.00428**
AURKA	7.215284	0.03781*	1.0552	2.873	1.435–5.75	0.00288**
OIP5	6.026397	0.05557	0.9789	2.662	1.374–5.156	0.00372**
CTHRC1	7.827664	0.0001814***	1.631	5.109	2.4–10.88	2.33e‐5***
GOLGA7B	7.534541	0.1695	1.1095	3.033	1.371–6.71	0.00617**

a Microarray data of SigMuc1NW's component genes were retrieved from the MSKCC dataset (cBioPortal).

b Cutpoint was estimated using Maximally Selected Rank Statistics in R.

c Coefficient to BCR was determined using univariate Cox proportion hazard analysis.

*P < 0.05; **P < 0.01; ***P < 0.001.

Finally, we made an attempt to compare the performance of SigMuc1NW to Prolaris (cell cycle progression/CPC) (Cuzick *et al*., 2011) in predicting BCR. The basis for this comparison was the similarities between SigMuc1NW to CPC: (a) like CPC, SigMuc1NW affects cell cycle progression (Table S2A and S2C; also see Discussion), and (b) similar to CPC, SigMuc1NW predicts BCR. As the CPC component genes promote cell cycle progression, we analyzed their effects on BCR using the 2 SD expression level. In the TCGA Provisional cohort, CPC is not correlated with a reduction in OS but significantly associated with BCR (Fig. S6). However, the predictive accuracy is lower than SigMuc1NW (comparing Fig. 2 and Fig. S6). Considering Prolaris being a real‐time PCR‐based signature and SigMuc1NW being derived from RNA seq, this comparison may not fully realize Prolaris effectiveness in predicting BCR. Nonetheless, it suggests that SigMuc1NW (Fig. 2A, MMDF 63.24, *P* = 1.12e‐12) offers comparable efficacy to Prolaris (Fig. S6, MMDF 66.89, *P* = 1.34e‐4) in assessing PC recurrence.

## 4 Discussion

Progression to biochemical recurrence is a major turning point in PC development; from there, a large proportion of PC will metastasize (Shipley *et al*., 2017), leading to ultimate death. The current treatments to metastatic PC are essentially palliative. It is thus highly desirable to effectively stratify PCs with higher risk of BCR following RP, allowing early intervention prior to metastatic progression.

MUC1 drives tumor progression in multiple tumor types (Kufe, 2009; de Paula Peres *et al*., 2015; Wurz *et al*., 2014) through activating important oncogenic proteins including EGFR, β‐catenin, NF‐κB, PKM2, and other pathways (Kufe, 2009; Singh and Hollingsworth, 2006; Wong *et al*., 2015). In line with its functions, a 9‐gene genomic signature was recently constructed from the MUC1 genomic network, which predicts BCR with a relatively good effectiveness (Lin *et al*., 2017). Using a novel system, we report here a robust improvement of this 9‐gene genomic signature in predicting BCR by systemically exploring its associated transcriptome. To our best knowledge, this is the first thorough analysis not on a single gene‐associated but rather on a multigene signature‐associated transcriptome consisting of 696 genes (Table S1). Because of the complex nature of cancer progression, in this case the progression to BCR, we chose not to focus on a specific aspect or pathway of tumorigenesis and instead performed a systemic examination of these 696 genes for their predictive power in BCR.

This novel and comprehensive analytic approach has resulted in a new 15‐gene panel. In the panel, 73.3% (11/15) of genes have not been reported to associate with PC. These 11 new PC genes include MGAT4B and OIP5. The former may play a role in the alteration of protein glycosylation, which is well known for being an important aspect of tumorigenesis (Munkley *et al*., 2016). Abnormalities in MUC1 glycosylation have been well demonstrated in tumorigenesis (Kufe, 2009; de Paula Peres *et al*., 2015). Thus, the inclusion of MGAT4B in the 15‐gene panel is in accordance with the panel being derived from a 9‐gene MUC1 genomic signature (Lin *et al*., 2017). The presence of OIP5 in SigMuc1NW suggests the protein as a tumor‐associated antigen (TAA) in PC. TAAs have been extensively investigated in cancer diagnosis and therapy (Scheid *et al*., 2016). In this regard, the OIP5's potential in PC diagnosis and therapy should be pursued.

As the construction of SigMuc1NW was not aimed on specific pathways, the gene panel covers multiple pathways. In addition to the potential effects on protein glycosylation though MGAT4B, the panel contains proteins with RNA helicase activity (SUPV3L1, Table 1) and DNA methyltransferase activity (DNMT3B, Table 1). These activities are important in gene expression and epigenetic alterations, which are well known to facilitate caner progression. SigMuc1NW also have a component of cell proliferation. AURKA is emerging as an important regulator of mitosis and a critical player in tumorigenesis. As such, AURKA is a hotly pursued in cancer therapy (Dominguez‐Brauer *et al*., 2015; Plotnikova *et al*., 2015). Additionally, OIP5 is also known as Mis18β which has recently been shown to play an important role in chromatid separation during mitosis (Nardi *et al*., 2016; Stellfox *et al*., 2016), adding another appealing feature for its inclusion in SigMuc1NW. Intriguingly, among the 15 genes, only four are known to function in PC and all four genes facilitate CRPC development, which is in accordance with the detection of SigMuc1NW elevation in mCRPCs (Table 6). As alterations in gene expression and the epigenetic patterns are involved in CRPC, the 15‐gene panel may also predict CRPC development, which will be examined in the future.

Inclusion of genes functioning in multiple pathways is likely a major attributor for the robust nature of the signature. SigMuc1NW and a set of its subsignatures all effectively stratify PC with increased risk of BCR with *P*‐value being the lowest (0) and are able to discriminate recurrent PC with tAUC >75%. Through combination of the subsignatures, sensitivity, specificity, and PPV can be achieved at high levels, 97.2%/, 98.1%, and 86.7% (Fig. 7B–E). Collectively, these evidences strongly indicate that the signatures constructed in this study will have important clinical applications in predicting PC recurrence.

This possible clinical application is supported by that the 15‐gene panel is likely not overfitted. (a) The overfitting issue is largely taken care of by modeling the 696 DEGs with covariate selection coupled with regularization (Elastic‐net penalty in R) with 10‐fold cross‐validation. (b) The component genes were directly examined using a different system: maximally selected rank statistics‐derived cutpoint; importantly, this system clearly improved the effectiveness of the SD‐based signature. (c) The signatures were robust in two independent PC cohorts (TCGA Provisional and MSKCC). (d) RNA was profiled through RNA sequencing (TCGA) and microarray analysis (MSKCC). (e) The 15‐gene panel is robustly associated with adverse feature of PC: Gleason scores and tumor stages. These associations likely resulted in the reduced HR of Gleason scores and tumor stages when they were analyzed with SigMuc1NW in multivariate Cox analysis (Table 3).

Between two commercially available multigene panels, Oncotype DX (12 genes plus 5 reference genes) and Prolaris (31 genes), there are no overlapping genes (Cuzick *et al*., 2011; Knezevic *et al*., 2013). This suggests the coexistence of different genesets with predictive values toward PC recurrence, which might be attributable to the complex mechanisms involved in disease progression. In this regard, our newly established SigMuc1NW, which contains a different set of genes from Oncotype DX and Prolaris, will enrich our ability to assess the risk of PC recurrence. While our research comprehensively supports that the signatures constructed here will have attractive clinical applications, realization of this potential requires further investigation.

## 5 Conclusions

We have formulated a novel strategy to derive differentially expressed genes (DEGs) relative to a reported PC signature from the most comprehensive and large PC genomic dataset (the TCGA dataset) and to systemically analyze these DEGs (*n* = 696) for pathways affected and impacts on PC recurrence. In this effort, a novel multigene set (*n* = 15 genes, SigMuc1NW) has been constructed. SigMuc1NW robustly predicts PC recurrence and is an independent risk factor of PC recurrence after adjusting for age at diagnosis, Gleason score, surgical margin, and tumor stage. Among these 15 component genes include 5 candidate oncogenic genes and 6 novel PC genes; within these 11 novel genes affecting PC recurrence, 6 genes (SLCO2A1, SUPV3L1, TATDN2, MGAT4B, SLC25A33, and OIP5) individually predict PC recurrence after adjusting for the above clinical factors. Collectively, we have identified novel genes affecting oncogenesis in general and PC pathogenesis in particular as well as constructed a novel and robust multigene set predicting PC recurrence using our system reported here. This system will have applications in exploration of publically available datasets for factors affecting cancer progression.

## Author contributions

YJ, WM, and DT performed literature search and initial analyses. YG, XL, LH, and HZ contributed to the analysis. FW, XW, HY, PM, and DT designed the research. PM and DT supervised the project. YJ, WM, YG, PM, and DT prepared the manuscript. All authors edited the manuscript and approved the final manuscript for submission.

## Supporting information


**Fig. S1.** Covariate selection from 696 DEGs using Elastic‐net penalty.

**Fig. S2.** Overlapping between the 9‐gene genomic signature which we have previously reported (Lin *et al*., 2017) and the current signature (SigMuc1NW). Graph was produced using the TCGA Provisional dataset (*n* = 492, cBioPortal).

**Fig. S3.** The combined signature is significantly associated with reductions in DFS and OS in PC patients.

**Fig. S4.** Cutpoint estimation.

**Fig. S5.** SigMuc1NW scores effectively stratify PCs with elevated risk of recurrence following RP.

**Fig. S6.** CPC geneset is associated with a reduction in DFS but not OS in PC patients.

**Table S1.** Differentially expression genes (DEGs) of a 9‐gene signature identified in the TCGA Provisional dataset.

**Table S2.** (A) Upregulation of gene sets among the 696 DEGs associated with the 9‐gene genomic signature within the kegg.sets.hs dataset. (B) Downregulation of gene sets among the 696 DEGs within the kegg.sets.hs dataset. (C) Upregulation of gene sets among the 696 DEGs within the GO.sets.hs dataset. (D) Downregulation of gene sets among the 696 DEGs within the GO.sets.hs dataset. (E) Pathways affected by the 696 DEGs associated with the 9‐gene genomic signature.

**Table S3.** Scores of the component genes and some clinical characteristics of patients with prostate cancer in the TCGA Provisional dataset within cBioPortal.

**Table S4.** Demographics of the TCGA patient population. The clinical characteristics were extracted from the TCGA Provisional dataset within cBioPortal along with the indicated clinical data.

**Table S5.** Cutpoints of individual gene expression determined by RNA sequencing.
