# Supplementary figures and images for: Construction of a set of novel and robust gene expression signatures predicting prostate cancer recurrence
Source: Mol Oncol. 2018 Aug 11;12(9):1559–78. doi: 10.1002/1878-0261.12359 (PMC6120243; doi:10.1002/1878-0261.12359)

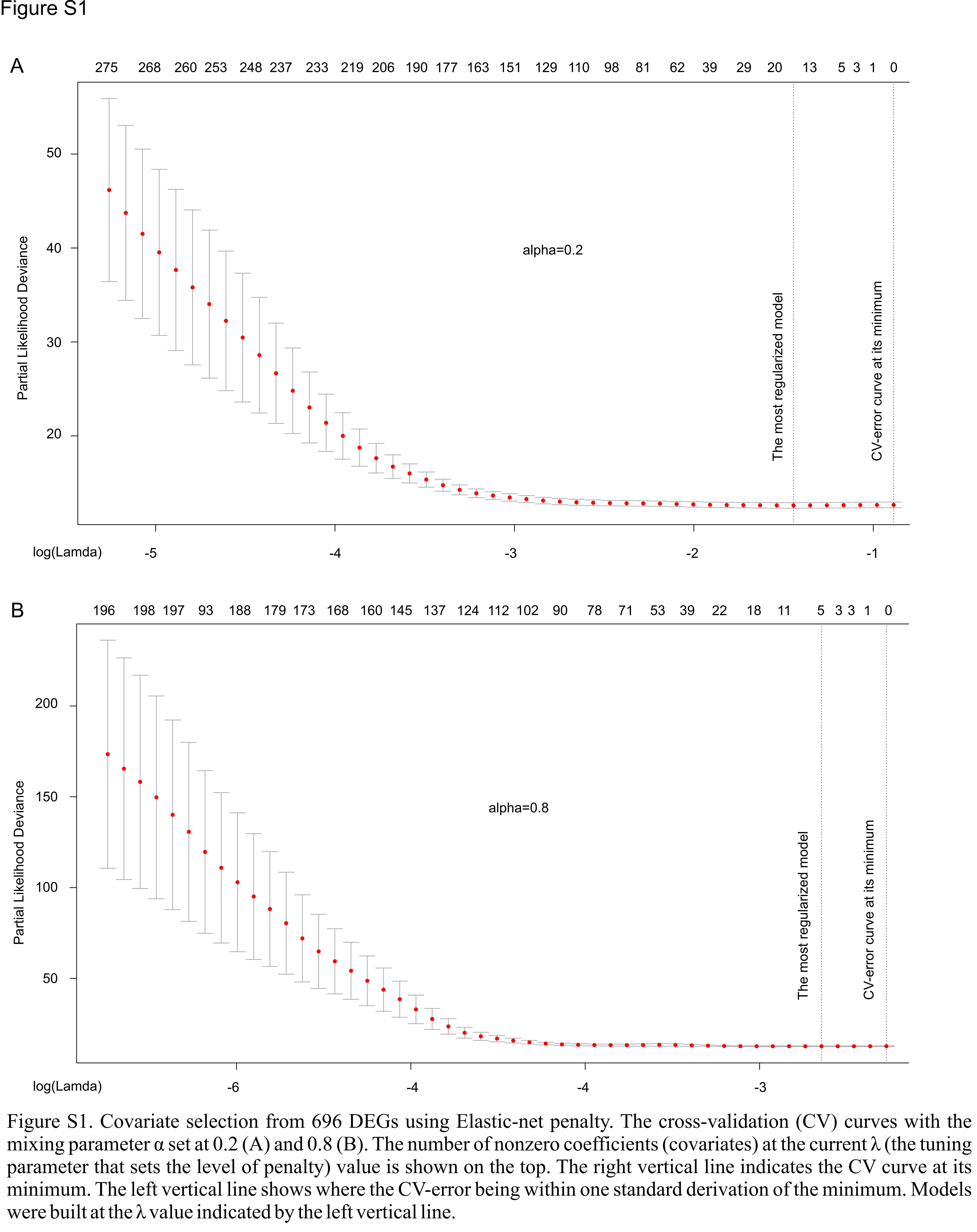

Supplement: Supplementary file 1 — Fig. S1. Covariate selection from 696 DEGs using Elastic‐net penalty. [file MOL2-12-1559-s001.tif]

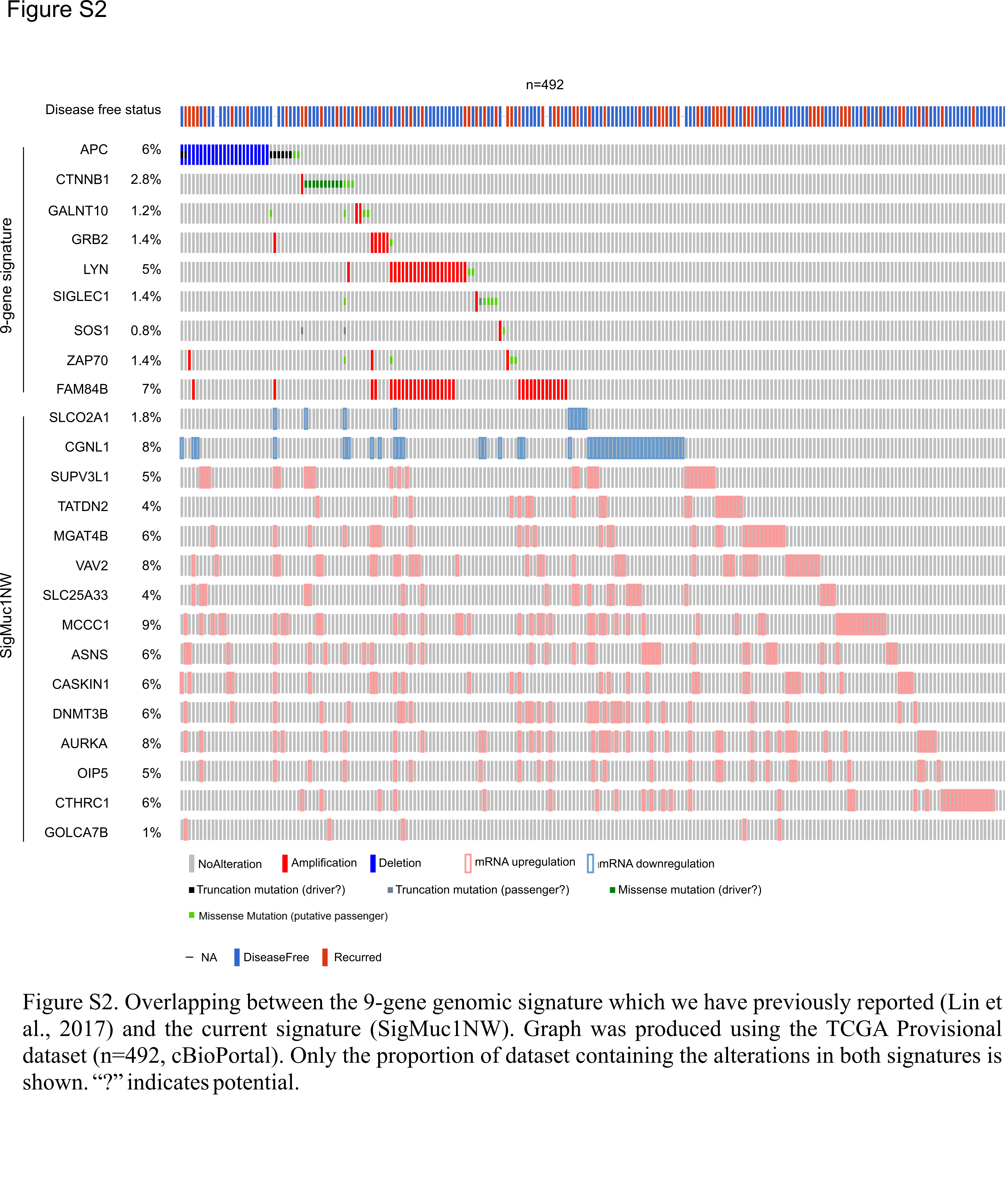

Supplement: Supplementary file 2 — Fig. S2. Overlapping between the 9‐gene genomic signature which we have previously reported (Lin et al., 2017) and the current signature (SigMuc1NW). Graph was produced using the TCGA Provisional dataset (n = 492, cBioPortal). [file MOL2-12-1559-s002.tif]

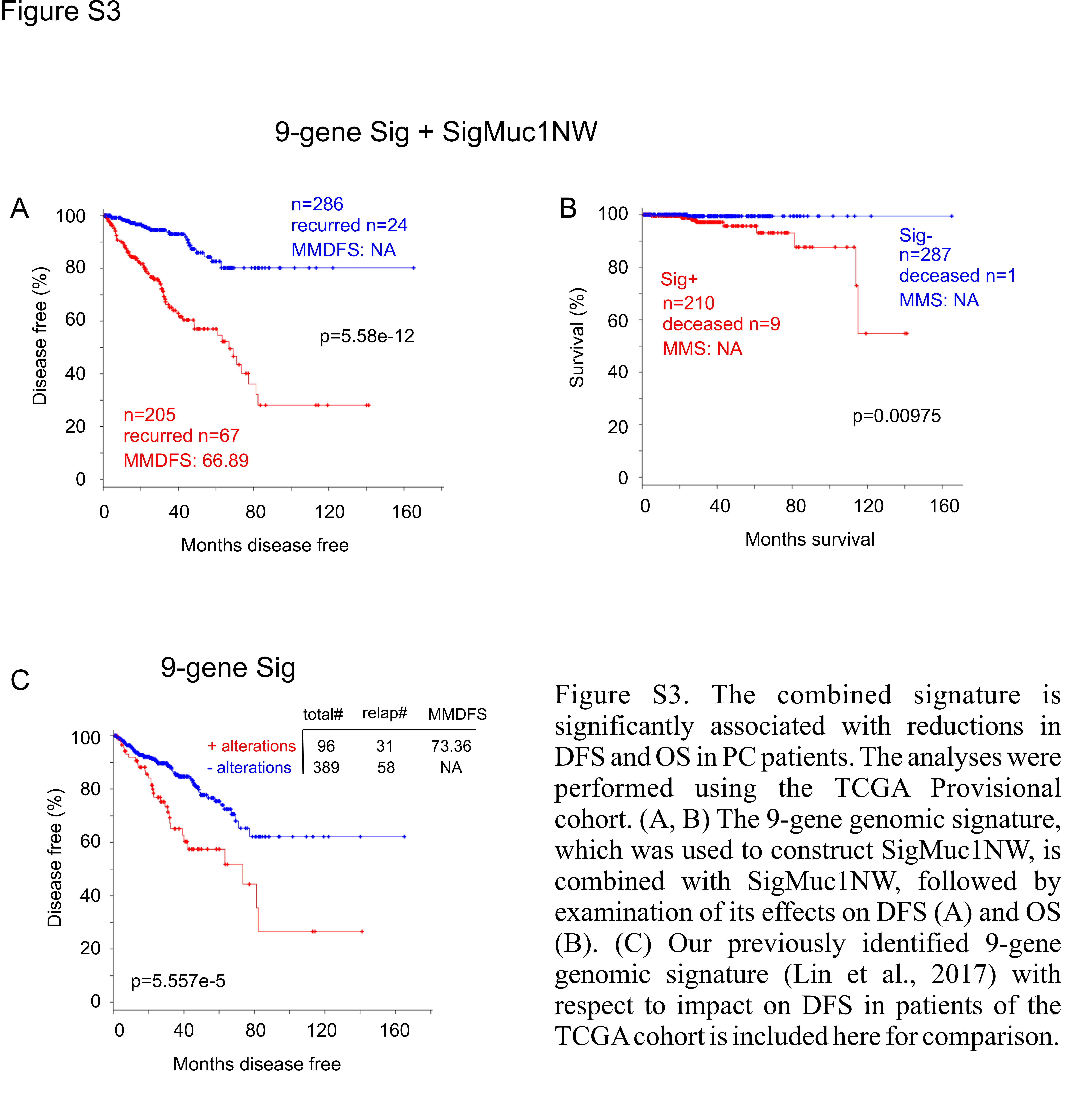

Supplement: Supplementary file 3 — Fig. S3. The combined signature is significantly associated with reductions in DFS and OS in PC patients. [file MOL2-12-1559-s003.tif]

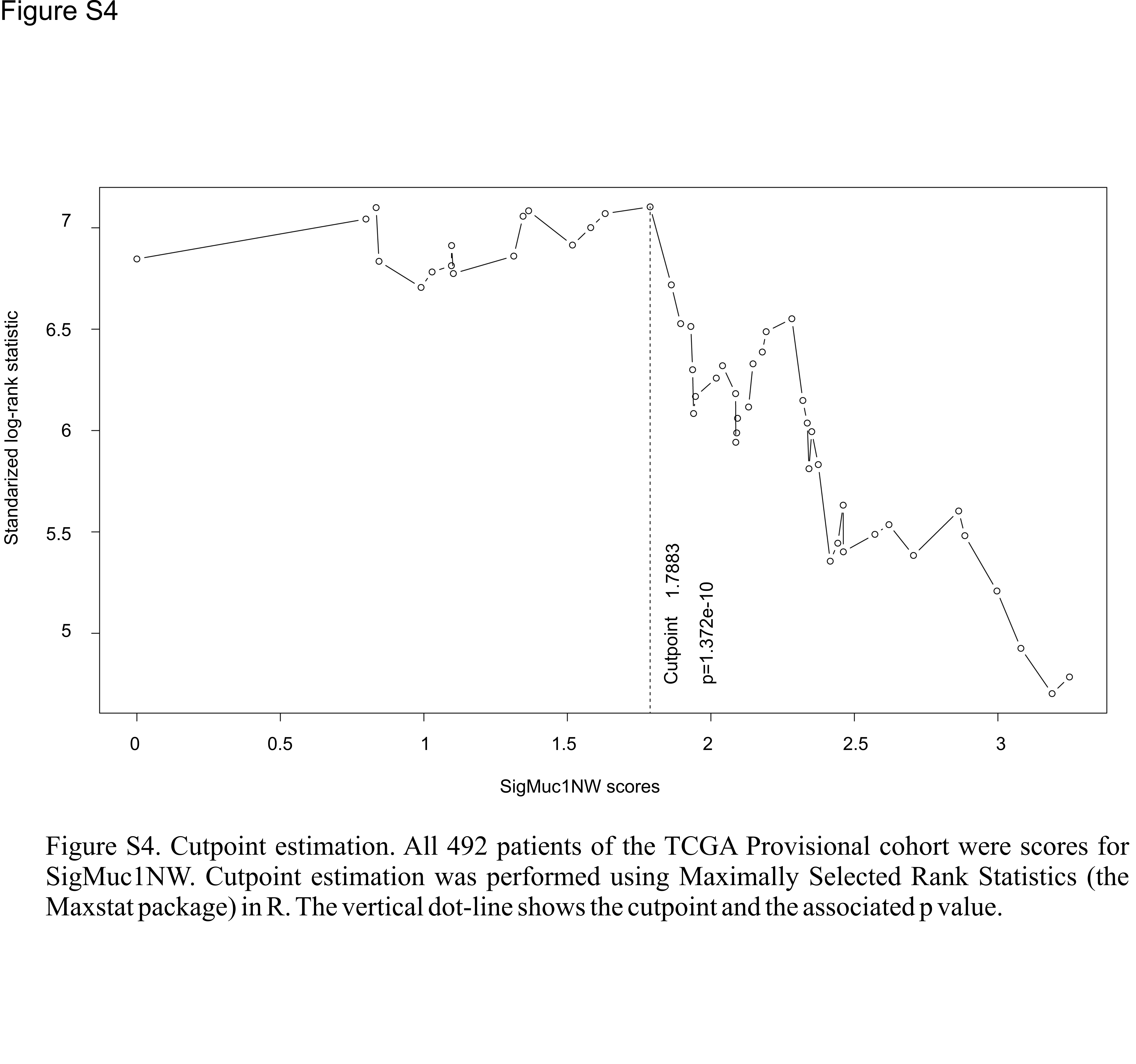

Supplement: Supplementary file 4 — Fig. S4. Cutpoint estimation. [file MOL2-12-1559-s004.tif]

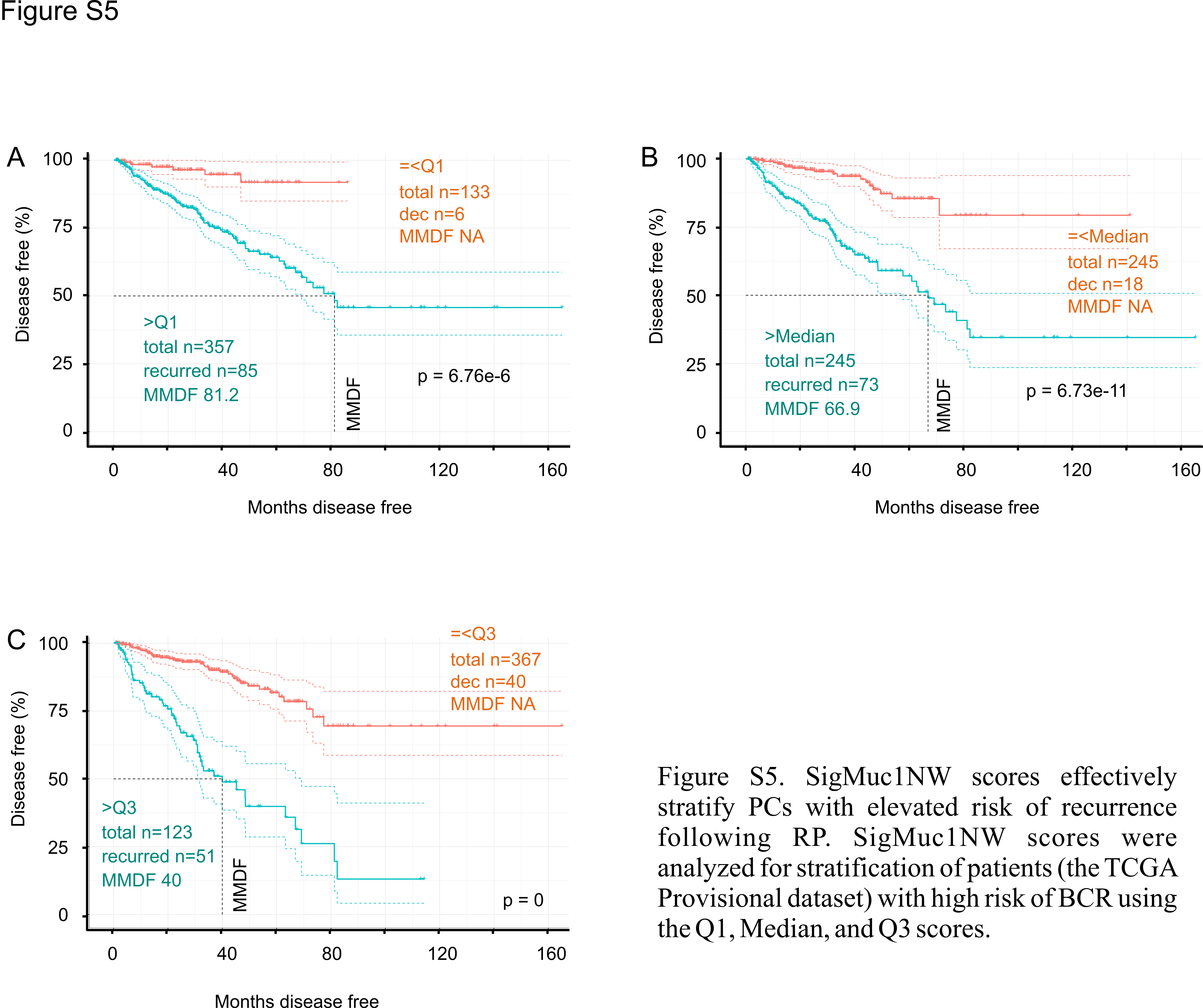

Supplement: Supplementary file 5 — Fig. S5. SigMuc1NW scores effectively stratify PCs with elevated risk of recurrence following RP. [file MOL2-12-1559-s005.tif]

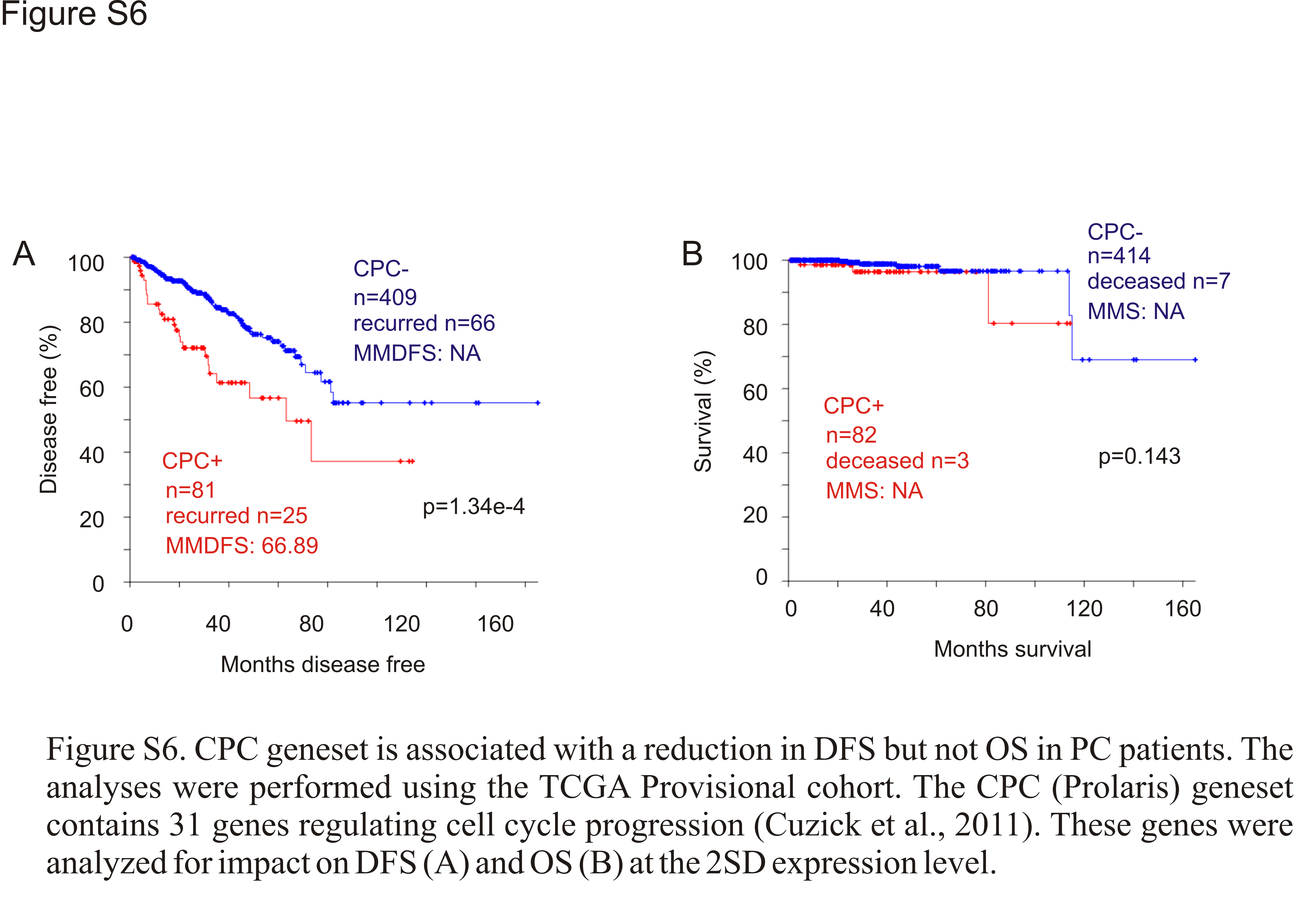

Supplement: Supplementary file 6 — Fig. S6. CPC geneset is associated with a reduction in DFS but not OS in PC patients. [file MOL2-12-1559-s006.tif]
