# Supplementary material for: Construction of a set of novel and robust gene expression signatures predicting prostate cancer recurrence
Source: Mol Oncol. 2018 Aug 11;12(9):1559–78. doi: 10.1002/1878-0261.12359 (PMC6120243; doi:10.1002/1878-0261.12359)
Supplement: Supplementary file 10 — Table S4. Demographics of the TCGA patient population. The clinical characteristics were extracted from the TCGA Provisional dataset within cBioPortal along with the indicated clinical data. [file MOL2-12-1559-s010.docx]

Table S4. Demographics of the TCGA patient population

| Characteristics | TCGA (n=490) | % |
| --- | --- | --- |
| Age (years)  Median (Q1-Q3) | 61 (56-66) | NA |
| ISUP  I  II  III  IV  V | 44  145  100  64  137 | 8.98%  29.59%  20.41%  13.06%  27.96% |
| Tumor stage  0 (T1 and T2)  1 (T3 and T4)  NA | 184  300  6 | 37.55%  61.22%  1.22% |
| Surgical margin  R0  R1  Rx  NA | 311  150  15  14 | 63.47%  30.61%  3.06%  2.86% |

Q: quartile

ISUP: International Society of Urological Pathology

NA: not available

R0: margin negative

R1: margin positive

Rx: margin not defined
